# Methyl 2-amino-4-(morpholin-4-yl)benzo[*d*]thiazole-6-carboxyl­ate tetartohydrate

**DOI:** 10.1107/S2414314624012495

**Published:** 2025-01-10

**Authors:** Erik Uran, Andrej Emanuel Cotman, Matic Lozinšek

**Affiliations:** a Jožef Stefan Institute, Jamova cesta 39, 1000 Ljubljana, Slovenia; b Jožef Stefan International Postgraduate School, Jamova cesta 39, 1000 Ljubljana, Slovenia; c Department of Pharmaceutical Chemistry, Faculty of Pharmacy, University of Ljubljana, Aškerčeva cesta 7, 1000 Ljubljana, Slovenia; University of Aberdeen, United Kingdom

**Keywords:** crystal structure, hydrogen bonds, chalcogen bonds, hydrate

## Abstract

The title compound, C_13_H_15_N_3_O_3_S·0.25H_2_O, crystallizes in the triclinic space group *P*1 and features four organic mol­ecules in the asymmetric unit alongside one water mol­ecule. The extended structure exhibits both hydrogen bonds (O—H⋯O, N—H⋯O and N—H⋯N) and chalcogen (C—S⋯O) contacts, leading to a complex three-dimensional network.

## Abstract 2

The crystal structure of a hydrate of the title benzo[*d*]thia­zole derivate is reported, which features a complex hydrogen-bonded network.

## Graphical abstract

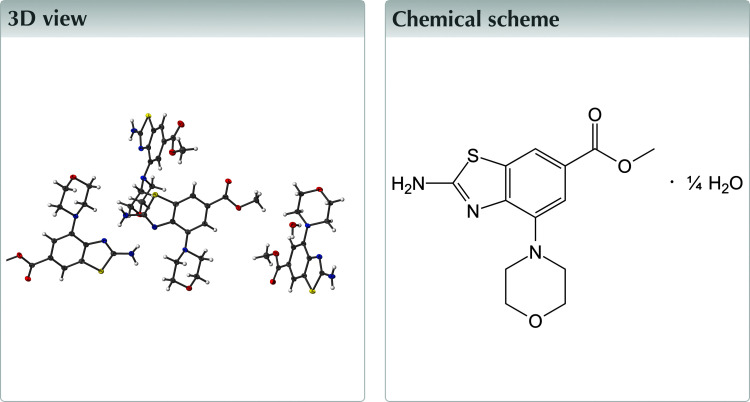

## Structure description

The discovery and development of anti­bacterials has been a critical focus in medicinal chemistry, with their significance growing due to the rise of bacterial resistance (Theuretzbacher *et al.*, 2020 ▸). To address this challenge, novel biologically active scaffolds have been explored in anti­bacterial development. Benzo­thia­zole-cored compounds featuring various substituents on the phenyl and thia­zole rings exhibit inhibitory effects on bacterial DNA gyrase and topoisomerase IV, key enzymes involved in bacterial DNA replication (Stokes *et al.*, 2013 ▸; Gjorgjieva *et al.*, 2016 ▸; Nyerges *et al.*, 2020 ▸; Cotman *et al.*, 2023 ▸; Durcik *et al.*, 2023 ▸). The title compound is one of the inter­mediates that was employed in the synthesis of the anti­bacterials with a 2-(1*H*-pyrrole-2-amido)­benzo[*d*]thia­zole scaffold (Durcik *et al.*, 2023 ▸).

**Figure 1:**
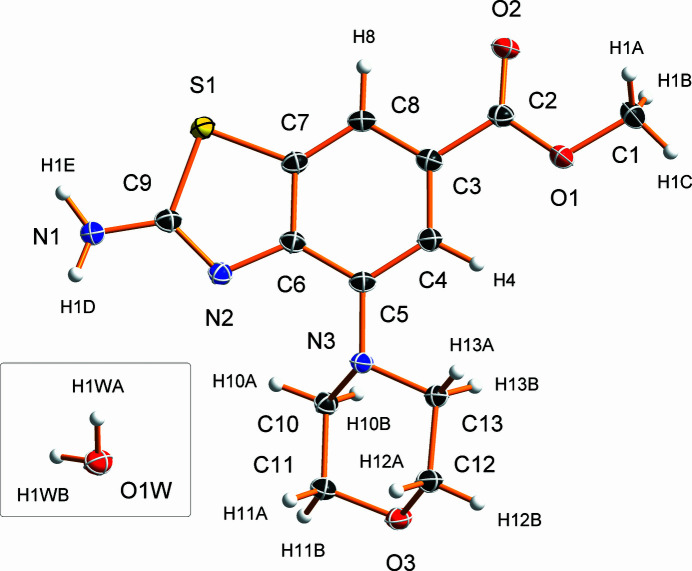
Water mol­ecule (insert) and one of the four crystallographically unique methyl 2-amino-4-morpholino­benzo[*d*]thia­zole-6-carboxyl­ate mol­ecules of the asymmetric unit of the title crystal structure and the corresponding atom-labelling scheme. Displacement ellipsoids are depicted at the 50% probability level and hydrogen atoms are shown as spheres of arbitrary radius.

The title hydrate crystallizes in the triclinic space group *P*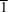 with *Z* = 2. The asymmetric unit is composed of four symmetry-independent methyl 2-amino-4-morpholino­benzo[*d*]thia­zole-6-carboxyl­ate (C_13_H_15_N_3_O_3_S) mol­ecules and a water mol­ecule of crystallization (Fig. 1 ▸).

**Figure 2:**
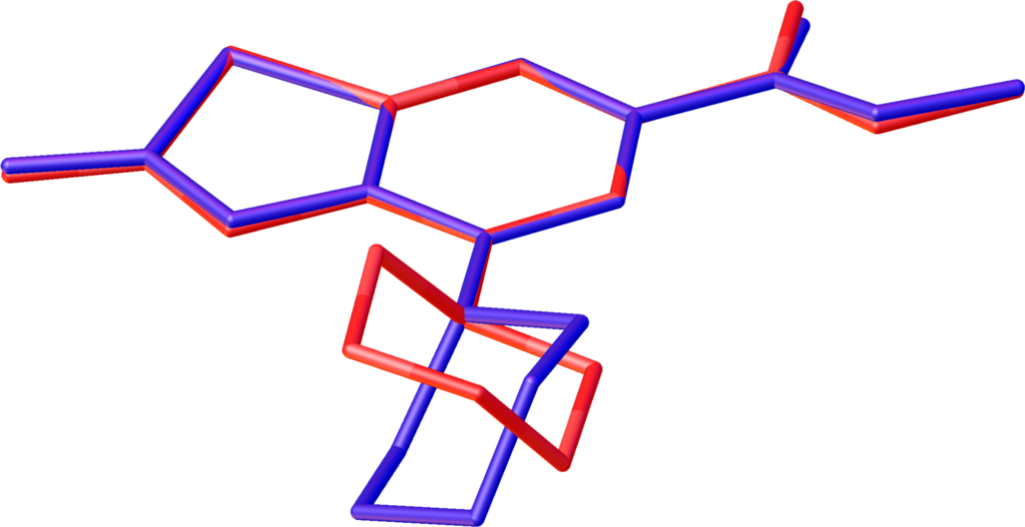
Mol­ecular overlap of two crystallographically independent methyl 2-amino-4-morpholino­benzo[*d*]thia­zole-6-carboxyl­ate mol­ecules S2 (red) and S3 (blue) with different orientations of their morpholine fragments. Hydrogen atoms are omitted for clarity.

In all the organic mol­ecules the –OOC– groups are slightly rotated around the OOC—C(Ph) bond with dihedral angles varying from 2.52 (7) to 10.94 (5)°. The terminal amino groups are positioned slightly out of the plane of the phenyl rings, with displacement values ranging from −0.304 (3) to 0.128 (3) Å. The C—NH_2_ distances [1.3371 (16)–1.3456 (16) Å] are shorter than the C—N distances between the benzene and morpholine rings [1.4204 (14)–1.4295 (14) Å]. The S—C bond lengths to the benzene ring [1.7400 (12)–1.7488 (12) Å] are shorter than the S—C distances in the S–C(NH_2_) moieties [1.7620 (12)–1.7679 (12) Å]. The morpholine fragment adopts a chair conformation and its orientation with respect to the benzene ring is nearly the same in three crystallographically independent mol­ecules containing S1, S3, and S4 [torsion angles C6—C5—N3—C10 = 59.59 (14)°, C32—C31—N9—C36 = 66.67 (14)°, C45—C44—N12—C49 = 67.40 (13)°], whereas in mol­ecule S2 it is different [C19—C18—N6—C23 = −57.61 (14)°] (Fig. 2 ▸).

**Figure 3:**
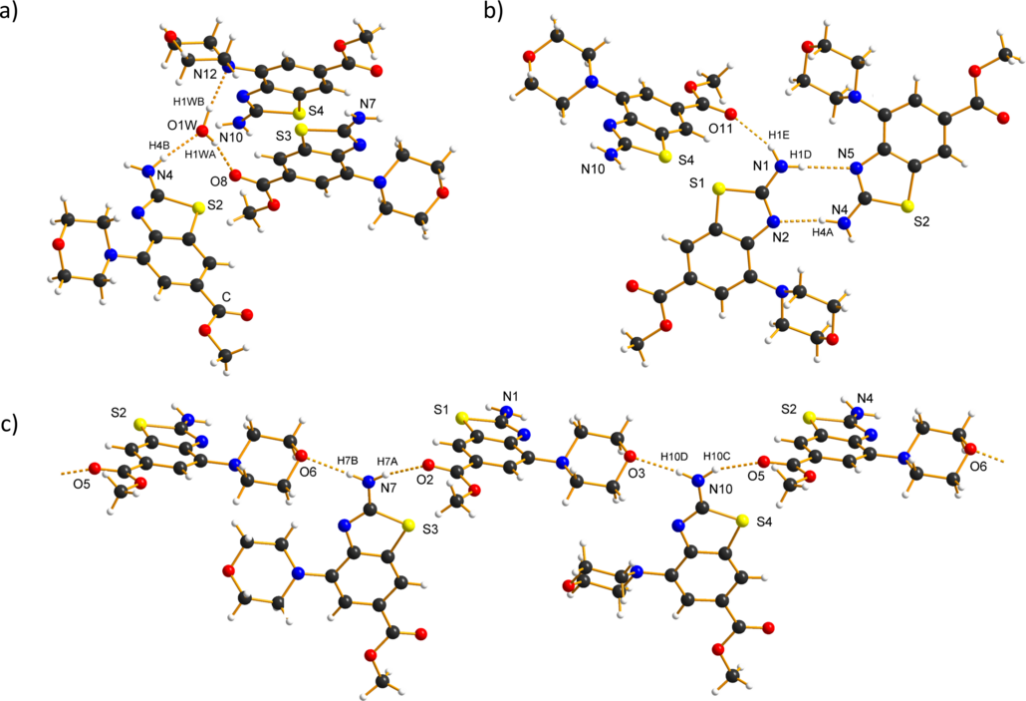
Hydrogen bonds in the title crystal structure (Table 1 ▸): (*a*) hydrogen bonding between the water mol­ecule and the three crystallographically independent organic mol­ecules; (*b*) hydrogen-bonded dimer with graph-set motif 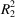
(8); (*c*) and hydrogen-bonded chain 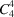
(24).

The water mol­ecule is hydrogen-bonded to three organic mol­ecules — as a hydrogen-bond donor to the morpholine nitro­gen atom of the S4 mol­ecule and the carbonyl oxygen atom of the ester group of the S3 mol­ecule and with the –NH_2_ group of the S2 mol­ecule as an acceptor (Fig. 3 ▸*a)*. In all the other hydrogen bonds (Table 1 ▸), the donors are the –NH_2_ groups (Fig. 3 ▸*b*,*c*). The acceptors are morpholine oxygen atoms, thia­zole nitro­gen atoms and the remaining three carbonyl oxygen atoms. The supra­molecular motifs observed in the crystal structure include a hydrogen-bonded dimer and a hydrogen-bonded chain composed of four crystallographically independent mol­ecules, with graph-set notation 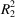
(8) and 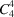
(24), respectively (Etter, 1990 ▸; Etter *et al.*, 1990 ▸).

As observed in certain sulfur-containing organic compounds, sulfur atoms can act as donors in chalcogen-bonding inter­actions (Scilabra *et al.*, 2019 ▸; Aakeroy *et al.*, 2019 ▸). A search of the Cambridge Structural Database (CSD v. 5.46, Nov. 2024; Groom *et al.*, 2016 ▸) was conducted to identify compounds containing a 1,3-benzo­thia­zole ring that participates in S⋯O contacts. The search criteria included: an S⋯O distance shorter than the sum of the van der Waals radii, a C—S⋯O angle in the range of 120–180°, and the selection of only organic structures with atomic coordinates and no errors. The search returned a subset of 256 entries for C_thia­zole_—S⋯O, with an average S⋯O distance of 3.186 ± 0.123 Å and an average C—S⋯O angle of 158 ± 16° and a second subset of 118 entries for C_phen­yl_—S⋯O, with an average S⋯O distance of 3.219 ± 0.129 Å and an average C—S⋯O angle of 158 ± 14°. One of the two C_phen­yl_—S⋯O contacts observed in the title crystal structure is slightly shorter [3.0600 (9) Å] and the other slightly longer [3.2336 (10) Å] than the average distance from CSD, with the former C_phen­yl_—S⋯O contact deviating more from linearity [162.76 (4)°] than the latter [170.31 (4)°] (Table 2 ▸).

**Figure 4:**
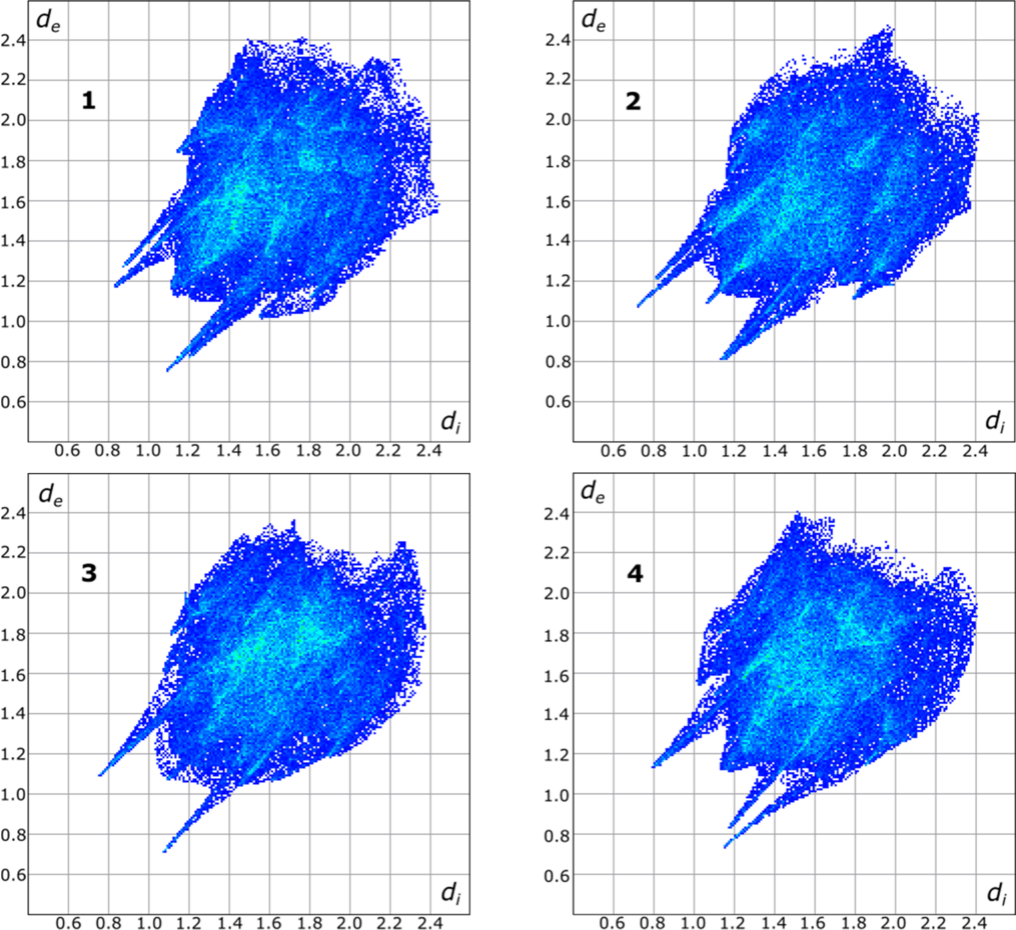
Hirshfeld two-dimensional fingerprint plots for the four crystallographically independent organic mol­ecules. The numbers 1, 2, 3 and 4 denote mol­ecules with sulfur atoms S1, S2, S3, and S4, respectively.

Hirshfeld two-dimensional fingerprint plots (Spackman & McKinnon, 2002 ▸; Spackman *et al.*, 2021 ▸) show that crystallographically independent mol­ecules of the asymmetric unit differ in their packing environments (Fig. 4 ▸). Overall, a complex three-dimensional network supported predominantly by hydrogen bonds is observed in the crystal structure.

## Synthesis and crystallization

The title compound was synthesized according to a modified literature procedure (Durcik *et al.*, 2023 ▸). Bromine (2.01 g, 12.5 mmol) was added to a solution of KSCN (2.44 g, 25.1 mmol) in glacial acetic acid (30 ml) and stirred at 25 °C for 30 min. The resulting mixture was added to a solution of methyl 4-amino-3-morpholino­benzoate (1.98 g, 8.37 mmol) in glacial acetic acid (20 ml) and the reaction mixture was stirred at 22 °C overnight. The resulting orange suspension was neutralized with 4 M NaOH(aq) until pH 8. The precipitate was collected and washed with water (30 ml). The filter cake was dried under reduced pressure and the residue was percolated with boiling methanol (5 × 20 ml). The filtrate was concentrated, and the solid residue was triturated with cold methanol (5 ml) to give the crude product (1.60 g). A 615 mg sample of the crude product was purified by column chromatography on silica using di­chloro­methane–methanol 20:1 as eluent (*R_f_* = 0.26). The fractions containing >99% of the product were combined and concentrated under reduced pressure to get the title compound as a white crystalline solid (272 mg, 29% yield). A suitable crystal was selected under the microscope and mounted on a MiTeGen Dual Thickness MicroLoop LD using Baysilone-Paste (Bayer-Silicone, mittelviskos).

## Refinement

Crystal data, data collection, and structure refinement details are summarized in Table 3 ▸. The positions of the hydrogen atoms were located from difference electron-density maps and refined freely, including their isotropic displacement parameter *U* (Cooper *et al.*, 2010 ▸).

## Supplementary Material

Crystal structure: contains datablock(s) I. DOI: 10.1107/S2414314624012495/hb4503sup1.cif

Structure factors: contains datablock(s) I. DOI: 10.1107/S2414314624012495/hb4503Isup2.hkl

Supporting information file. DOI: 10.1107/S2414314624012495/hb4503Isup3.cml

CCDC reference: 2413286

Additional supporting information:  crystallographic information; 3D view; checkCIF report

## Figures and Tables

**Table 1:** Hydrogen-bond geometry (Å, °)

*D*—H⋯*A*	*D*—H	H⋯*A*	*D*⋯*A*	*D*—H⋯*A*
N1—H1*D*⋯N5	0.844 (18)	2.324 (18)	3.1642 (15)	174.3 (16)
N1—H1*E*⋯O11^i^	0.876 (18)	2.137 (18)	2.9475 (14)	153.6 (15)
N4—H4*A*⋯N2	0.90 (2)	2.13 (2)	2.9881 (15)	159.0 (16)
N4—H4*B*⋯O1*W*^ii^	0.858 (19)	1.957 (19)	2.8038 (14)	168.8 (17)
N7—H7*A*⋯O2^iii^	0.840 (19)	2.024 (19)	2.8556 (14)	170.8 (17)
N7—H7*B*⋯O6^iv^	0.857 (19)	2.120 (19)	2.9625 (14)	167.5 (17)
N10—H10*C*⋯O5^v^	0.870 (18)	2.088 (18)	2.9101 (14)	157.3 (15)
N10—H10*D*⋯O3^ii^	0.839 (19)	2.11 (2)	2.9479 (14)	172.6 (17)
O1*W*—H1*WA*⋯O8^v^	0.93 (2)	1.84 (2)	2.7451 (13)	162.6 (19)
O1*W*—H1*WB*⋯N12	0.97 (2)	1.91 (2)	2.8669 (13)	169 (2)

Symmetry codes: (i) 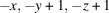; (ii) 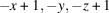; (iii) 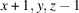; (iv) 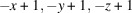; (v) 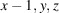.

**Table 2:** Geometry of C—S⋯O chalcogen contacts present in the crystal structure (Å, °)

C—S⋯O	C—S	S⋯O	C—S⋯O	Nc(S⋯O)^*a*^/Nc(S⋯O)^*b*^
C7—S1⋯O11	1.7400 (12)	3.0600 (9)	162.76 (4)	0.90 / 0.92
C46—S4⋯O5	1.7440 (12)	3.2336 (10)	170.31 (4)	0.95 / 0.97

Nc(S⋯O) = *d*(S⋯O)/[*r*_vdW_(S) + *r*_vdW_(O)]; the normalized contact, Nc, (Scilabra *et al.*, 2019 ▸) is the ratio of the experimental S⋯O distance to the sum of S and O van der Waals radii *r*_vdW_(S) and *r*_vdW_(O): (*a*) 1.89 Å and 1.50 Å (Alvarez, 2013 ▸) and (*b*) 1.80 Å and 1.52 Å (Bondi, 1964 ▸).

**Table 3:** Experimental details

Crystal data	
Chemical formula	4C_13_H_15_N_3_O_3_S·H_2_O
*M*_r_	1191.37
Crystal system, space group	Triclinic, *P* 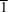
Temperature (K)	100
*a*, *b*, *c* (Å)	12.47742 (16), 15.1037 (2), 15.7031 (2)
α, β, γ (°)	75.3037 (14), 72.5571 (13), 71.7565 (13)
*V* (Å^3^)	2639.29 (7)
*Z*	2
Radiation type	Cu *K*α
μ (mm^−1^)	2.32
Crystal size (mm)	0.25 × 0.18 × 0.03

Data collection	
Diffractometer	XtaLAB Synergy, Dualflex, Eiger2 R CdTe 1M
Absorption correction	Gaussian (*CrysAlis PRO*; Rigaku OD, 2023 ▸)
*T*_min_, *T*_max_	0.240, 1.000
No. of measured, independent and observed [*I* > 2σ(*I*)] reflections	114974, 10968, 10229
*R*_int_	0.037
(sin θ/λ)_max_ (Å^−1^)	0.630

Refinement	
*R*[*F*^2^ > 2σ(*F*^2^)], *wR*(*F*^2^), *S*	0.031, 0.087, 1.07
No. of reflections	10968
No. of parameters	978
H-atom treatment	All H-atom parameters refined
Δρ_max_, Δρ_min_ (e Å^−3^)	0.45, −0.33

Computer programs: *CrysAlis PRO* (Rigaku OD, 2023 ▸), *OLEX2.solve* (Bourhis *et al.*, 2015 ▸), *OLEX2* (Dolomanov *et al.*, 2009 ▸), *SHELXL* (Sheldrick, 2015 ▸), *DIAMOND* (Brandenburg, 2005 ▸), *Crystal Explorer 17* (Spackman *et al.*, 2021 ▸) and *publCIF* (Westrip, 2010 ▸).

## full crystallographic data

### Methyl 2-amino-4-(morpholin-4-yl)benzo[d]thiazole-6-carboxylate tetartohydrate Crystal data

**Table:**

4C_13_H_15_N_3_O_3_S·H_2_O	*Z* = 2
*M**_r_* = 1191.37	*F*(000) = 1252
Triclinic, *P*1	*D*_x_ = 1.499 Mg m^−^^3^
*a* = 12.47742 (16) Å	Cu *K*α radiation, λ = 1.54184 Å
*b* = 15.1037 (2) Å	Cell parameters from 69685 reflections
*c* = 15.7031 (2) Å	θ = 3.0–75.8°
α = 75.3037 (14)°	µ = 2.32 mm^−^^1^
β = 72.5571 (13)°	*T* = 100 K
γ = 71.7565 (13)°	Plate, clear colourless
*V* = 2639.29 (7) Å^3^	0.25 × 0.18 × 0.03 mm

### Methyl 2-amino-4-(morpholin-4-yl)benzo[d]thiazole-6-carboxylate tetartohydrate Data collection

**Table:**

XtaLAB Synergy, Dualflex, Eiger2 1M diffractometer	10968 independent reflections
Radiation source: micro-focus sealed X-ray tube, PhotonJet (Cu) X-ray Source	10229 reflections with *I* > 2σ(*I*)
Mirror monochromator	*R*_int_ = 0.037
Detector resolution: 13.3333 pixels mm^-1^	θ_max_ = 76.2°, θ_min_ = 3.0°
ω scans	*h* = −15→15
Absorption correction: gaussian (CrysAlisPro; Rigaku OD, 2023)	*k* = −18→18
*T*_min_ = 0.240, *T*_max_ = 1.000	*l* = −17→19
114974 measured reflections	

### Methyl 2-amino-4-(morpholin-4-yl)benzo[d]thiazole-6-carboxylate tetartohydrate Refinement

**Table:**

Refinement on *F*^2^	Primary atom site location: dual
Least-squares matrix: full	Hydrogen site location: difference Fourier map
*R*[*F*^2^ > 2σ(*F*^2^)] = 0.031	All H-atom parameters refined
*wR*(*F*^2^) = 0.087	*w* = 1/[σ^2^(*F*_o_^2^) + (0.0532*P*)^2^ + 0.892*P*] where *P* = (*F*_o_^2^ + 2*F*_c_^2^)/3
*S* = 1.07	(Δ/σ)_max_ = 0.001
10968 reflections	Δρ_max_ = 0.45 e Å^−^^3^
978 parameters	Δρ_min_ = −0.33 e Å^−^^3^
0 restraints	

### Methyl 2-amino-4-(morpholin-4-yl)benzo[d]thiazole-6-carboxylate tetartohydrate Special details

**Table:**

Geometry. All esds (except the esd in the dihedral angle between two l.s. planes) are estimated using the full covariance matrix. The cell esds are taken into account individually in the estimation of esds in distances, angles and torsion angles; correlations between esds in cell parameters are only used when they are defined by crystal symmetry. An approximate (isotropic) treatment of cell esds is used for estimating esds involving l.s. planes.

### Methyl 2-amino-4-(morpholin-4-yl)benzo[d]thiazole-6-carboxylate tetartohydrate Fractional atomic coordinates and isotropic or equivalent isotropic displacement parameters (Å^2^)

**Table:**

	*x*	*y*	*z*	*U*_iso_*/*U*_eq_	
C1	0.54589 (13)	0.36920 (10)	1.31862 (9)	0.0271 (3)	
H1A	0.5533 (14)	0.4299 (13)	1.3140 (11)	0.028 (4)*	
H1B	0.4735 (16)	0.3612 (13)	1.3584 (13)	0.036 (5)*	
H1C	0.6098 (15)	0.3215 (13)	1.3398 (12)	0.034 (4)*	
C2	0.47041 (10)	0.40797 (9)	1.18989 (8)	0.0186 (2)	
C3	0.48132 (10)	0.38683 (8)	1.10017 (8)	0.0184 (2)	
C4	0.57960 (10)	0.32158 (8)	1.05928 (8)	0.0179 (2)	
H4	0.6389 (13)	0.2929 (11)	1.0900 (10)	0.020 (4)*	
C5	0.59078 (10)	0.30065 (8)	0.97499 (8)	0.0163 (2)	
C6	0.49719 (10)	0.34536 (8)	0.93244 (8)	0.0166 (2)	
C7	0.40222 (10)	0.41315 (8)	0.97364 (8)	0.0176 (2)	
C8	0.39231 (10)	0.43495 (8)	1.05669 (8)	0.0186 (2)	
H8	0.3271 (14)	0.4797 (11)	1.0861 (11)	0.024 (4)*	
C9	0.39001 (10)	0.37965 (8)	0.83270 (8)	0.0181 (2)	
C10	0.74956 (10)	0.27903 (8)	0.84191 (8)	0.0176 (2)	
H10A	0.6956 (13)	0.3064 (11)	0.8048 (11)	0.021 (4)*	
H10B	0.7788 (13)	0.3312 (11)	0.8487 (10)	0.020 (4)*	
C11	0.85121 (10)	0.20591 (9)	0.79758 (8)	0.0197 (2)	
H11A	0.8241 (13)	0.1579 (11)	0.7842 (10)	0.020 (4)*	
H11B	0.8950 (13)	0.2367 (11)	0.7397 (10)	0.019 (4)*	
C12	0.87326 (11)	0.11550 (9)	0.93859 (8)	0.0200 (2)	
H12A	0.8441 (14)	0.0677 (12)	0.9277 (11)	0.026 (4)*	
H12B	0.9297 (13)	0.0868 (11)	0.9724 (10)	0.020 (4)*	
C13	0.77449 (10)	0.18661 (8)	0.98778 (8)	0.0179 (2)	
H13A	0.8058 (13)	0.2319 (11)	1.0047 (10)	0.021 (4)*	
H13B	0.7353 (13)	0.1533 (11)	1.0444 (10)	0.019 (3)*	
N1	0.35455 (10)	0.37659 (8)	0.76082 (7)	0.0217 (2)	
H1D	0.3893 (15)	0.3326 (12)	0.7306 (12)	0.026 (4)*	
H1E	0.2830 (16)	0.4076 (12)	0.7583 (11)	0.029 (4)*	
N2	0.48765 (8)	0.32704 (7)	0.85314 (7)	0.01689 (19)	
N3	0.69018 (8)	0.23587 (7)	0.93242 (7)	0.01607 (19)	
O1	0.55373 (8)	0.35088 (6)	1.23066 (6)	0.02321 (19)	
O2	0.39368 (8)	0.47031 (7)	1.22433 (6)	0.02458 (19)	
O3	0.93240 (7)	0.16173 (6)	0.85408 (6)	0.02016 (18)	
S1	0.30117 (2)	0.45796 (2)	0.90732 (2)	0.01871 (7)	
C14	0.45617 (12)	0.09901 (11)	0.20550 (9)	0.0265 (3)	
H14A	0.5039 (15)	0.1342 (12)	0.1604 (12)	0.032 (4)*	
H14B	0.4880 (15)	0.0317 (13)	0.2084 (11)	0.030 (4)*	
H14C	0.3805 (17)	0.1161 (13)	0.1978 (13)	0.040 (5)*	
C15	0.53289 (10)	0.08947 (8)	0.32837 (8)	0.0184 (2)	
C16	0.51177 (10)	0.11878 (8)	0.41637 (8)	0.0182 (2)	
C17	0.40644 (10)	0.18031 (8)	0.45346 (8)	0.0175 (2)	
H17	0.3478 (13)	0.2012 (11)	0.4204 (10)	0.021 (4)*	
C18	0.38726 (10)	0.20914 (8)	0.53573 (8)	0.0168 (2)	
C19	0.47919 (10)	0.17594 (8)	0.58090 (8)	0.0171 (2)	
C20	0.58036 (10)	0.10954 (8)	0.54461 (8)	0.0183 (2)	
C21	0.59913 (10)	0.08139 (8)	0.46283 (8)	0.0192 (2)	
H21	0.6704 (16)	0.0382 (13)	0.4390 (12)	0.035 (5)*	
C22	0.57412 (10)	0.15158 (8)	0.68705 (8)	0.0179 (2)	
C23	0.22453 (10)	0.22890 (9)	0.66662 (8)	0.0181 (2)	
H23A	0.2017 (13)	0.1714 (11)	0.6653 (10)	0.019 (3)*	
H23B	0.2783 (14)	0.2099 (11)	0.7050 (11)	0.023 (4)*	
C24	0.11721 (10)	0.30087 (9)	0.70638 (8)	0.0198 (2)	
H24A	0.0757 (13)	0.2697 (10)	0.7667 (10)	0.017 (3)*	
H24B	0.1373 (13)	0.3576 (11)	0.7143 (10)	0.021 (4)*	
C25	0.08901 (11)	0.37590 (9)	0.56173 (8)	0.0201 (2)	
H25A	0.1090 (13)	0.4316 (11)	0.5658 (10)	0.019 (3)*	
H25B	0.0309 (13)	0.3944 (11)	0.5272 (10)	0.020 (4)*	
C26	0.19608 (10)	0.30632 (9)	0.51784 (8)	0.0186 (2)	
H26A	0.2330 (13)	0.3394 (11)	0.4592 (11)	0.022 (4)*	
H26B	0.1708 (13)	0.2544 (11)	0.5055 (10)	0.022 (4)*	
N4	0.60343 (9)	0.16044 (8)	0.75911 (7)	0.0197 (2)	
H4A	0.5557 (16)	0.2015 (13)	0.7954 (13)	0.037 (5)*	
H4B	0.6541 (16)	0.1132 (13)	0.7797 (12)	0.032 (4)*	
N5	0.47893 (9)	0.20100 (7)	0.66030 (7)	0.0175 (2)	
N6	0.28050 (8)	0.26995 (7)	0.57439 (6)	0.0166 (2)	
O4	0.44174 (8)	0.12563 (7)	0.29108 (6)	0.02308 (19)	
O5	0.62317 (8)	0.03788 (7)	0.29295 (6)	0.02431 (19)	
O6	0.03542 (7)	0.33291 (6)	0.65044 (6)	0.02030 (18)	
S2	0.67429 (2)	0.07262 (2)	0.61666 (2)	0.01892 (7)	
C27	0.89000 (12)	−0.07898 (9)	0.44252 (10)	0.0245 (3)	
H27A	0.9620 (14)	−0.1268 (11)	0.4495 (10)	0.021 (4)*	
H27B	0.8756 (14)	−0.0781 (12)	0.3843 (12)	0.031 (4)*	
H27C	0.8216 (16)	−0.0893 (13)	0.4957 (12)	0.037 (5)*	
C28	0.98949 (11)	0.04040 (9)	0.38065 (8)	0.0199 (2)	
C29	0.99220 (11)	0.13827 (9)	0.37925 (8)	0.0197 (2)	
C30	0.89719 (11)	0.19927 (9)	0.42839 (8)	0.0194 (2)	
H30	0.8306 (13)	0.1769 (11)	0.4635 (11)	0.022 (4)*	
C31	0.89710 (10)	0.29203 (9)	0.42660 (8)	0.0185 (2)	
C32	0.99572 (10)	0.32369 (8)	0.37216 (8)	0.0173 (2)	
C33	1.09087 (10)	0.26045 (9)	0.32574 (8)	0.0184 (2)	
C34	1.09116 (11)	0.16791 (9)	0.32777 (8)	0.0193 (2)	
H34	1.1568 (14)	0.1260 (11)	0.2966 (11)	0.022 (4)*	
C35	1.11095 (10)	0.42025 (8)	0.31257 (8)	0.0179 (2)	
C36	0.74244 (11)	0.43866 (9)	0.42929 (8)	0.0203 (2)	
H36A	0.6959 (14)	0.4207 (11)	0.3967 (11)	0.026 (4)*	
H36B	0.7991 (13)	0.4695 (11)	0.3828 (11)	0.021 (4)*	
C37	0.66191 (11)	0.50517 (9)	0.49337 (9)	0.0251 (3)	
H37A	0.7098 (14)	0.5266 (12)	0.5211 (11)	0.027 (4)*	
H37B	0.6126 (13)	0.5605 (11)	0.4596 (10)	0.022 (4)*	
C38	0.65108 (12)	0.37953 (9)	0.61433 (9)	0.0245 (3)	
H38A	0.5953 (13)	0.3505 (11)	0.6637 (10)	0.021 (4)*	
H38B	0.7017 (14)	0.4007 (12)	0.6405 (11)	0.029 (4)*	
C39	0.72681 (11)	0.30808 (9)	0.55398 (9)	0.0229 (3)	
H39A	0.6762 (13)	0.2855 (11)	0.5282 (10)	0.022 (4)*	
H39B	0.7726 (14)	0.2538 (11)	0.5896 (11)	0.025 (4)*	
N7	1.14804 (10)	0.49939 (8)	0.29154 (8)	0.0217 (2)	
H7A	1.2186 (16)	0.4970 (12)	0.2683 (12)	0.031 (4)*	
H7B	1.1026 (16)	0.5491 (13)	0.3120 (12)	0.032 (4)*	
N8	1.00881 (9)	0.41355 (7)	0.36339 (7)	0.0179 (2)	
N9	0.80725 (9)	0.35308 (7)	0.48018 (7)	0.0195 (2)	
O7	0.89986 (8)	0.01373 (6)	0.44346 (6)	0.02269 (18)	
O8	1.05888 (8)	−0.01186 (6)	0.33071 (6)	0.0259 (2)	
O9	0.58498 (8)	0.45893 (6)	0.56481 (6)	0.0258 (2)	
S3	1.20204 (2)	0.31714 (2)	0.27013 (2)	0.01850 (7)	
C40	0.06370 (13)	0.59803 (9)	0.08511 (10)	0.0245 (3)	
H40A	0.0722 (14)	0.6021 (12)	0.1440 (12)	0.030 (4)*	
H40B	0.1277 (16)	0.6123 (13)	0.0354 (13)	0.039 (5)*	
H40C	−0.0044 (16)	0.6397 (13)	0.0724 (12)	0.034 (4)*	
C41	−0.02630 (10)	0.47168 (8)	0.13882 (8)	0.0185 (2)	
C42	−0.01716 (10)	0.37236 (8)	0.13491 (8)	0.0175 (2)	
C43	0.08502 (10)	0.31725 (8)	0.08605 (8)	0.0172 (2)	
H43	0.1477 (13)	0.3456 (10)	0.0569 (10)	0.016 (3)*	
C44	0.09484 (10)	0.22410 (8)	0.08173 (8)	0.0161 (2)	
C45	−0.00167 (10)	0.18614 (8)	0.12692 (8)	0.0163 (2)	
C46	−0.10314 (10)	0.24303 (8)	0.17501 (8)	0.0173 (2)	
C47	−0.11225 (10)	0.33536 (8)	0.18068 (8)	0.0181 (2)	
H47	−0.1807 (14)	0.3715 (11)	0.2151 (11)	0.022 (4)*	
C48	−0.10401 (10)	0.07990 (8)	0.18043 (8)	0.0172 (2)	
C49	0.19192 (11)	0.13321 (9)	−0.04116 (8)	0.0219 (2)	
H49A	0.1251 (14)	0.1067 (11)	−0.0254 (10)	0.022 (4)*	
H49B	0.1830 (14)	0.1899 (12)	−0.0918 (11)	0.028 (4)*	
C50	0.30243 (11)	0.05804 (10)	−0.07130 (9)	0.0249 (3)	
H50A	0.2995 (14)	0.0427 (11)	−0.1257 (12)	0.028 (4)*	
H50B	0.3095 (13)	−0.0003 (11)	−0.0226 (11)	0.023 (4)*	
C51	0.41042 (10)	0.12163 (9)	−0.01560 (8)	0.0203 (2)	
H51A	0.4182 (12)	0.0657 (11)	0.0341 (10)	0.018 (3)*	
H51B	0.4825 (13)	0.1445 (10)	−0.0348 (10)	0.019 (3)*	
C52	0.30376 (10)	0.19959 (8)	0.01456 (9)	0.0189 (2)	
H52A	0.2986 (13)	0.2544 (11)	−0.0357 (11)	0.025 (4)*	
H52B	0.3098 (13)	0.2189 (11)	0.0668 (11)	0.022 (4)*	
N10	−0.13303 (9)	−0.00242 (8)	0.19632 (7)	0.0205 (2)	
H10C	−0.2003 (16)	−0.0070 (12)	0.2319 (12)	0.028 (4)*	
H10D	−0.0800 (16)	−0.0510 (13)	0.1843 (12)	0.033 (4)*	
N11	−0.00422 (8)	0.09423 (7)	0.13051 (7)	0.0171 (2)	
N12	0.19992 (8)	0.16389 (7)	0.03829 (7)	0.01621 (19)	
O10	0.06595 (8)	0.50184 (6)	0.08508 (6)	0.02260 (18)	
O11	−0.10817 (8)	0.52123 (6)	0.18464 (6)	0.02325 (19)	
O12	0.40343 (7)	0.09221 (7)	−0.09280 (6)	0.02370 (19)	
S4	−0.20530 (2)	0.17707 (2)	0.22683 (2)	0.01769 (7)	
O1W	0.24165 (8)	0.00903 (7)	0.18409 (6)	0.02453 (19)	
H1WA	0.1704 (19)	0.0078 (15)	0.2254 (15)	0.055 (6)*	
H1WB	0.224 (2)	0.0561 (17)	0.1316 (17)	0.067 (7)*	

### Methyl 2-amino-4-(morpholin-4-yl)benzo[d]thiazole-6-carboxylate tetartohydrate Atomic displacement parameters (Å^2^)

**Table:**

	*U*^11^	*U*^22^	*U*^33^	*U*^12^	*U*^13^	*U*^23^
C1	0.0313 (7)	0.0305 (7)	0.0199 (6)	−0.0025 (6)	−0.0070 (5)	−0.0111 (5)
C2	0.0156 (5)	0.0197 (6)	0.0198 (6)	−0.0049 (4)	−0.0012 (4)	−0.0056 (4)
C3	0.0181 (6)	0.0179 (5)	0.0184 (6)	−0.0046 (4)	−0.0017 (4)	−0.0052 (4)
C4	0.0166 (5)	0.0174 (5)	0.0183 (6)	−0.0031 (4)	−0.0034 (4)	−0.0037 (4)
C5	0.0156 (5)	0.0137 (5)	0.0175 (5)	−0.0031 (4)	−0.0016 (4)	−0.0028 (4)
C6	0.0167 (5)	0.0157 (5)	0.0158 (5)	−0.0049 (4)	−0.0010 (4)	−0.0026 (4)
C7	0.0164 (5)	0.0151 (5)	0.0191 (6)	−0.0032 (4)	−0.0030 (4)	−0.0018 (4)
C8	0.0158 (5)	0.0165 (5)	0.0209 (6)	−0.0016 (4)	−0.0014 (4)	−0.0055 (4)
C9	0.0173 (5)	0.0171 (5)	0.0166 (5)	−0.0043 (4)	−0.0011 (4)	−0.0012 (4)
C10	0.0163 (5)	0.0188 (5)	0.0153 (5)	−0.0037 (4)	−0.0022 (4)	−0.0019 (4)
C11	0.0176 (5)	0.0231 (6)	0.0173 (6)	−0.0045 (5)	−0.0017 (4)	−0.0054 (5)
C12	0.0181 (6)	0.0187 (6)	0.0195 (6)	−0.0012 (5)	−0.0024 (5)	−0.0038 (5)
C13	0.0170 (5)	0.0183 (5)	0.0165 (6)	−0.0011 (4)	−0.0037 (4)	−0.0043 (4)
N1	0.0182 (5)	0.0266 (6)	0.0187 (5)	−0.0003 (4)	−0.0053 (4)	−0.0068 (4)
N2	0.0156 (4)	0.0181 (5)	0.0156 (5)	−0.0041 (4)	−0.0025 (4)	−0.0024 (4)
N3	0.0145 (4)	0.0169 (5)	0.0143 (5)	−0.0012 (4)	−0.0018 (4)	−0.0039 (4)
O1	0.0244 (4)	0.0250 (4)	0.0186 (4)	0.0012 (4)	−0.0062 (3)	−0.0094 (3)
O2	0.0193 (4)	0.0284 (5)	0.0253 (5)	−0.0003 (4)	−0.0023 (4)	−0.0146 (4)
O3	0.0149 (4)	0.0221 (4)	0.0199 (4)	−0.0021 (3)	−0.0015 (3)	−0.0041 (3)
S1	0.01674 (14)	0.01793 (14)	0.01871 (14)	0.00021 (10)	−0.00457 (11)	−0.00391 (11)
C14	0.0263 (7)	0.0358 (8)	0.0182 (6)	−0.0054 (6)	−0.0036 (5)	−0.0115 (5)
C15	0.0169 (5)	0.0190 (5)	0.0184 (6)	−0.0055 (4)	−0.0008 (4)	−0.0045 (4)
C16	0.0179 (6)	0.0179 (5)	0.0174 (6)	−0.0045 (4)	−0.0012 (4)	−0.0047 (4)
C17	0.0172 (5)	0.0173 (5)	0.0166 (5)	−0.0034 (4)	−0.0030 (4)	−0.0033 (4)
C18	0.0167 (5)	0.0151 (5)	0.0162 (5)	−0.0030 (4)	−0.0016 (4)	−0.0027 (4)
C19	0.0190 (5)	0.0164 (5)	0.0147 (5)	−0.0056 (4)	−0.0019 (4)	−0.0021 (4)
C20	0.0166 (5)	0.0177 (5)	0.0187 (6)	−0.0041 (4)	−0.0026 (4)	−0.0023 (4)
C21	0.0177 (6)	0.0182 (5)	0.0198 (6)	−0.0029 (4)	−0.0021 (4)	−0.0054 (4)
C22	0.0181 (5)	0.0169 (5)	0.0165 (5)	−0.0056 (4)	−0.0015 (4)	−0.0011 (4)
C23	0.0179 (6)	0.0203 (6)	0.0144 (5)	−0.0041 (5)	−0.0022 (4)	−0.0031 (4)
C24	0.0181 (6)	0.0242 (6)	0.0163 (6)	−0.0042 (5)	−0.0022 (4)	−0.0061 (5)
C25	0.0200 (6)	0.0193 (6)	0.0171 (6)	−0.0001 (5)	−0.0032 (5)	−0.0040 (5)
C26	0.0189 (6)	0.0190 (6)	0.0156 (6)	−0.0009 (5)	−0.0034 (4)	−0.0050 (4)
N4	0.0197 (5)	0.0203 (5)	0.0176 (5)	−0.0023 (4)	−0.0053 (4)	−0.0036 (4)
N5	0.0178 (5)	0.0190 (5)	0.0149 (5)	−0.0048 (4)	−0.0029 (4)	−0.0028 (4)
N6	0.0161 (5)	0.0179 (5)	0.0132 (5)	−0.0014 (4)	−0.0019 (4)	−0.0036 (4)
O4	0.0211 (4)	0.0291 (5)	0.0184 (4)	−0.0002 (4)	−0.0043 (3)	−0.0115 (4)
O5	0.0185 (4)	0.0296 (5)	0.0235 (5)	−0.0024 (4)	−0.0001 (3)	−0.0129 (4)
O6	0.0164 (4)	0.0235 (4)	0.0185 (4)	−0.0025 (3)	−0.0024 (3)	−0.0051 (3)
S2	0.01739 (14)	0.01890 (14)	0.01883 (14)	−0.00077 (11)	−0.00486 (11)	−0.00474 (11)
C27	0.0297 (7)	0.0193 (6)	0.0264 (7)	−0.0078 (5)	−0.0089 (5)	−0.0028 (5)
C28	0.0239 (6)	0.0200 (6)	0.0156 (5)	−0.0038 (5)	−0.0065 (5)	−0.0030 (4)
C29	0.0254 (6)	0.0181 (6)	0.0160 (6)	−0.0047 (5)	−0.0069 (5)	−0.0024 (4)
C30	0.0214 (6)	0.0202 (6)	0.0159 (5)	−0.0063 (5)	−0.0036 (5)	−0.0018 (4)
C31	0.0192 (6)	0.0199 (6)	0.0149 (5)	−0.0035 (4)	−0.0040 (4)	−0.0027 (4)
C32	0.0201 (6)	0.0170 (5)	0.0138 (5)	−0.0029 (4)	−0.0052 (4)	−0.0023 (4)
C33	0.0195 (6)	0.0203 (6)	0.0141 (5)	−0.0039 (5)	−0.0040 (4)	−0.0023 (4)
C34	0.0216 (6)	0.0183 (6)	0.0160 (6)	−0.0011 (5)	−0.0050 (5)	−0.0041 (4)
C35	0.0180 (5)	0.0184 (5)	0.0155 (5)	−0.0017 (4)	−0.0043 (4)	−0.0032 (4)
C36	0.0193 (6)	0.0200 (6)	0.0190 (6)	−0.0037 (5)	−0.0035 (5)	−0.0018 (5)
C37	0.0224 (6)	0.0204 (6)	0.0267 (7)	−0.0046 (5)	0.0017 (5)	−0.0040 (5)
C38	0.0263 (6)	0.0235 (6)	0.0193 (6)	−0.0065 (5)	0.0019 (5)	−0.0047 (5)
C39	0.0239 (6)	0.0207 (6)	0.0193 (6)	−0.0057 (5)	0.0009 (5)	−0.0027 (5)
N7	0.0159 (5)	0.0181 (5)	0.0277 (6)	−0.0027 (4)	−0.0001 (4)	−0.0062 (4)
N8	0.0180 (5)	0.0179 (5)	0.0164 (5)	−0.0035 (4)	−0.0035 (4)	−0.0030 (4)
N9	0.0199 (5)	0.0179 (5)	0.0161 (5)	−0.0031 (4)	−0.0002 (4)	−0.0023 (4)
O7	0.0246 (4)	0.0181 (4)	0.0244 (5)	−0.0064 (3)	−0.0031 (4)	−0.0043 (3)
O8	0.0319 (5)	0.0218 (4)	0.0220 (4)	−0.0067 (4)	−0.0010 (4)	−0.0072 (4)
O9	0.0215 (4)	0.0235 (4)	0.0258 (5)	−0.0043 (4)	0.0028 (4)	−0.0048 (4)
S3	0.01702 (14)	0.01802 (14)	0.01781 (14)	−0.00252 (11)	−0.00096 (11)	−0.00493 (11)
C40	0.0311 (7)	0.0163 (6)	0.0269 (7)	−0.0055 (5)	−0.0085 (6)	−0.0044 (5)
C41	0.0193 (6)	0.0178 (6)	0.0179 (6)	−0.0005 (4)	−0.0078 (4)	−0.0037 (4)
C42	0.0197 (6)	0.0160 (5)	0.0157 (5)	−0.0006 (4)	−0.0060 (4)	−0.0036 (4)
C43	0.0185 (5)	0.0168 (5)	0.0145 (5)	−0.0028 (4)	−0.0039 (4)	−0.0021 (4)
C44	0.0160 (5)	0.0171 (5)	0.0123 (5)	−0.0005 (4)	−0.0031 (4)	−0.0028 (4)
C45	0.0170 (5)	0.0161 (5)	0.0132 (5)	−0.0006 (4)	−0.0040 (4)	−0.0022 (4)
C46	0.0155 (5)	0.0189 (6)	0.0143 (5)	−0.0016 (4)	−0.0027 (4)	−0.0023 (4)
C47	0.0176 (5)	0.0185 (6)	0.0148 (5)	0.0008 (4)	−0.0036 (4)	−0.0045 (4)
C48	0.0161 (5)	0.0192 (5)	0.0136 (5)	−0.0011 (4)	−0.0036 (4)	−0.0029 (4)
C49	0.0185 (6)	0.0287 (6)	0.0183 (6)	−0.0045 (5)	−0.0013 (5)	−0.0096 (5)
C50	0.0185 (6)	0.0315 (7)	0.0259 (7)	−0.0060 (5)	0.0015 (5)	−0.0159 (6)
C51	0.0170 (6)	0.0199 (6)	0.0217 (6)	−0.0030 (5)	−0.0011 (5)	−0.0061 (5)
C52	0.0161 (5)	0.0174 (5)	0.0209 (6)	−0.0033 (4)	−0.0012 (4)	−0.0044 (5)
N10	0.0150 (5)	0.0179 (5)	0.0241 (5)	−0.0023 (4)	0.0010 (4)	−0.0048 (4)
N11	0.0164 (5)	0.0161 (5)	0.0160 (5)	−0.0016 (4)	−0.0025 (4)	−0.0029 (4)
N12	0.0152 (5)	0.0159 (4)	0.0152 (5)	−0.0025 (4)	−0.0004 (4)	−0.0045 (4)
O10	0.0258 (4)	0.0159 (4)	0.0241 (4)	−0.0049 (3)	−0.0021 (4)	−0.0053 (3)
O11	0.0204 (4)	0.0216 (4)	0.0276 (5)	0.0008 (3)	−0.0062 (4)	−0.0114 (4)
O12	0.0184 (4)	0.0299 (5)	0.0206 (4)	−0.0063 (4)	0.0033 (3)	−0.0100 (4)
S4	0.01439 (13)	0.01794 (14)	0.01703 (14)	−0.00138 (10)	0.00000 (10)	−0.00467 (10)
O1W	0.0219 (4)	0.0240 (5)	0.0223 (5)	−0.0017 (4)	−0.0038 (4)	−0.0020 (4)

### Methyl 2-amino-4-(morpholin-4-yl)benzo[d]thiazole-6-carboxylate tetartohydrate Geometric parameters (Å, º)

**Table:**

C1—H1A	0.933 (18)	C27—H27B	0.979 (18)
C1—H1B	0.955 (19)	C27—H27C	1.020 (18)
C1—H1C	0.971 (18)	C27—O7	1.4477 (15)
C1—O1	1.4456 (15)	C28—C29	1.4836 (16)
C2—C3	1.4803 (17)	C28—O7	1.3435 (15)
C2—O1	1.3386 (15)	C28—O8	1.2110 (16)
C2—O2	1.2162 (15)	C29—C30	1.4065 (17)
C3—C4	1.4070 (16)	C29—C34	1.3938 (17)
C3—C8	1.3876 (17)	C30—H30	0.956 (16)
C4—H4	0.935 (16)	C30—C31	1.3939 (17)
C4—C5	1.3947 (17)	C31—C32	1.4157 (16)
C5—C6	1.4181 (16)	C31—N9	1.4126 (15)
C5—N3	1.4204 (14)	C32—C33	1.4062 (16)
C6—C7	1.4099 (16)	C32—N8	1.3850 (15)
C6—N2	1.3878 (15)	C33—C34	1.3887 (17)
C7—C8	1.3857 (17)	C33—S3	1.7488 (12)
C7—S1	1.7400 (12)	C34—H34	0.950 (16)
C8—H8	0.960 (16)	C35—N7	1.3440 (16)
C9—N1	1.3456 (16)	C35—N8	1.3044 (15)
C9—N2	1.3105 (15)	C35—S3	1.7679 (12)
C9—S1	1.7620 (12)	C36—H36A	1.009 (16)
C10—H10A	0.951 (16)	C36—H36B	0.987 (16)
C10—H10B	1.005 (15)	C36—C37	1.5186 (17)
C10—C11	1.5155 (16)	C36—N9	1.4739 (15)
C10—N3	1.4816 (15)	C37—H37A	1.000 (17)
C11—H11A	0.978 (16)	C37—H37B	1.003 (16)
C11—H11B	0.994 (15)	C37—O9	1.4298 (15)
C11—O3	1.4401 (14)	C38—H38A	0.987 (15)
C12—H12A	0.973 (16)	C38—H38B	1.010 (17)
C12—H12B	0.939 (16)	C38—C39	1.5162 (17)
C12—C13	1.5135 (16)	C38—O9	1.4195 (16)
C12—O3	1.4348 (14)	C39—H39A	1.019 (16)
C13—H13A	1.006 (16)	C39—H39B	0.989 (16)
C13—H13B	0.974 (15)	C39—N9	1.4637 (15)
C13—N3	1.4649 (15)	N7—H7A	0.840 (19)
N1—H1D	0.844 (18)	N7—H7B	0.857 (19)
N1—H1E	0.876 (18)	C40—H40A	0.981 (18)
C14—H14A	0.946 (18)	C40—H40B	0.970 (19)
C14—H14B	0.963 (18)	C40—H40C	0.928 (18)
C14—H14C	0.935 (19)	C40—O10	1.4445 (15)
C14—O4	1.4436 (15)	C41—C42	1.4840 (16)
C15—C16	1.4811 (16)	C41—O10	1.3399 (15)
C15—O4	1.3351 (15)	C41—O11	1.2145 (15)
C15—O5	1.2166 (15)	C42—C43	1.4072 (16)
C16—C17	1.4075 (16)	C42—C47	1.3916 (17)
C16—C21	1.3862 (17)	C43—H43	0.949 (15)
C17—H17	0.950 (15)	C43—C44	1.3908 (16)
C17—C18	1.3967 (17)	C44—C45	1.4127 (16)
C18—C19	1.4204 (16)	C44—N12	1.4295 (14)
C18—N6	1.4208 (14)	C45—C46	1.4095 (16)
C19—C20	1.4106 (16)	C45—N11	1.3850 (15)
C19—N5	1.3907 (15)	C46—C47	1.3869 (17)
C20—C21	1.3854 (17)	C46—S4	1.7440 (12)
C20—S2	1.7407 (12)	C47—H47	0.949 (16)
C21—H21	0.957 (18)	C48—N10	1.3451 (16)
C22—N4	1.3371 (16)	C48—N11	1.3074 (15)
C22—N5	1.3150 (15)	C48—S4	1.7639 (12)
C22—S2	1.7645 (12)	C49—H49A	0.976 (15)
C23—H23A	1.001 (15)	C49—H49B	1.013 (17)
C23—H23B	0.964 (16)	C49—C50	1.5218 (17)
C23—C24	1.5154 (16)	C49—N12	1.4759 (15)
C23—N6	1.4760 (15)	C50—H50A	0.954 (17)
C24—H24A	1.010 (15)	C50—H50B	1.010 (16)
C24—H24B	1.009 (16)	C50—O12	1.4256 (15)
C24—O6	1.4352 (14)	C51—H51A	0.996 (15)
C25—H25A	0.972 (15)	C51—H51B	0.999 (15)
C25—H25B	0.964 (15)	C51—C52	1.5186 (16)
C25—C26	1.5168 (16)	C51—O12	1.4266 (15)
C25—O6	1.4366 (14)	C52—H52A	0.990 (16)
C26—H26A	0.983 (16)	C52—H52B	0.967 (16)
C26—H26B	1.011 (16)	C52—N12	1.4678 (15)
C26—N6	1.4666 (15)	N10—H10C	0.870 (18)
N4—H4A	0.90 (2)	N10—H10D	0.839 (19)
N4—H4B	0.858 (19)	O1W—H1WA	0.93 (2)
C27—H27A	0.976 (16)	O1W—H1WB	0.97 (2)
			
H1A—C1—H1B	110.9 (15)	H27A—C27—H27C	112.1 (13)
H1A—C1—H1C	110.8 (14)	H27B—C27—H27C	112.3 (14)
H1B—C1—H1C	110.0 (15)	O7—C27—H27A	110.2 (9)
O1—C1—H1A	110.9 (10)	O7—C27—H27B	108.7 (10)
O1—C1—H1B	109.1 (11)	O7—C27—H27C	104.4 (10)
O1—C1—H1C	105.0 (10)	O7—C28—C29	113.26 (10)
O1—C2—C3	113.26 (10)	O8—C28—C29	125.02 (11)
O2—C2—C3	124.17 (11)	O8—C28—O7	121.72 (11)
O2—C2—O1	122.57 (11)	C30—C29—C28	120.72 (11)
C4—C3—C2	121.29 (11)	C34—C29—C28	118.15 (11)
C8—C3—C2	117.78 (11)	C34—C29—C30	121.13 (11)
C8—C3—C4	120.91 (11)	C29—C30—H30	119.3 (9)
C3—C4—H4	117.9 (9)	C31—C30—C29	121.62 (11)
C5—C4—C3	121.90 (11)	C31—C30—H30	119.0 (9)
C5—C4—H4	120.2 (9)	C30—C31—C32	117.62 (11)
C4—C5—C6	117.57 (10)	C30—C31—N9	123.23 (11)
C4—C5—N3	122.48 (10)	N9—C31—C32	119.05 (11)
C6—C5—N3	119.95 (10)	C33—C32—C31	119.58 (11)
C7—C6—C5	118.90 (11)	N8—C32—C31	124.52 (11)
N2—C6—C5	125.88 (10)	N8—C32—C33	115.84 (10)
N2—C6—C7	115.20 (10)	C32—C33—S3	109.19 (9)
C6—C7—S1	109.63 (9)	C34—C33—C32	122.76 (11)
C8—C7—C6	123.25 (11)	C34—C33—S3	128.04 (10)
C8—C7—S1	127.05 (9)	C29—C34—H34	121.3 (9)
C3—C8—H8	118.7 (10)	C33—C34—C29	117.23 (11)
C7—C8—C3	117.30 (11)	C33—C34—H34	121.4 (9)
C7—C8—H8	124.0 (10)	N7—C35—S3	120.12 (9)
N1—C9—S1	119.50 (9)	N8—C35—N7	123.92 (11)
N2—C9—N1	124.67 (11)	N8—C35—S3	115.96 (9)
N2—C9—S1	115.83 (9)	H36A—C36—H36B	107.5 (12)
H10A—C10—H10B	107.8 (12)	C37—C36—H36A	109.9 (9)
C11—C10—H10A	110.3 (9)	C37—C36—H36B	110.9 (9)
C11—C10—H10B	109.1 (9)	N9—C36—H36A	110.1 (9)
N3—C10—H10A	109.5 (9)	N9—C36—H36B	108.3 (9)
N3—C10—H10B	109.3 (9)	N9—C36—C37	110.10 (10)
N3—C10—C11	110.87 (9)	C36—C37—H37A	108.9 (10)
C10—C11—H11A	110.7 (9)	C36—C37—H37B	110.0 (9)
C10—C11—H11B	110.3 (9)	H37A—C37—H37B	111.0 (13)
H11A—C11—H11B	108.2 (12)	O9—C37—C36	111.33 (10)
O3—C11—C10	111.06 (10)	O9—C37—H37A	108.3 (9)
O3—C11—H11A	110.2 (9)	O9—C37—H37B	107.3 (9)
O3—C11—H11B	106.3 (9)	H38A—C38—H38B	109.9 (13)
H12A—C12—H12B	110.3 (13)	C39—C38—H38A	109.7 (9)
C13—C12—H12A	110.4 (10)	C39—C38—H38B	109.4 (10)
C13—C12—H12B	110.3 (9)	O9—C38—H38A	107.0 (9)
O3—C12—H12A	109.8 (9)	O9—C38—H38B	110.0 (9)
O3—C12—H12B	105.3 (9)	O9—C38—C39	110.80 (11)
O3—C12—C13	110.75 (10)	C38—C39—H39A	109.9 (9)
C12—C13—H13A	110.1 (9)	C38—C39—H39B	109.5 (9)
C12—C13—H13B	109.0 (9)	H39A—C39—H39B	110.0 (13)
H13A—C13—H13B	106.5 (12)	N9—C39—C38	108.82 (10)
N3—C13—C12	110.31 (10)	N9—C39—H39A	110.0 (9)
N3—C13—H13A	112.1 (9)	N9—C39—H39B	108.6 (9)
N3—C13—H13B	108.6 (9)	C35—N7—H7A	120.0 (12)
C9—N1—H1D	120.3 (11)	C35—N7—H7B	119.1 (12)
C9—N1—H1E	117.2 (11)	H7A—N7—H7B	119.6 (17)
H1D—N1—H1E	119.0 (16)	C35—N8—C32	110.48 (10)
C9—N2—C6	110.55 (10)	C31—N9—C36	115.39 (10)
C5—N3—C10	113.02 (9)	C31—N9—C39	115.99 (10)
C5—N3—C13	114.51 (9)	C39—N9—C36	110.34 (10)
C13—N3—C10	110.00 (9)	C28—O7—C27	114.69 (10)
C2—O1—C1	114.91 (10)	C38—O9—C37	109.36 (10)
C12—O3—C11	108.44 (9)	C33—S3—C35	88.53 (6)
C7—S1—C9	88.72 (6)	H40A—C40—H40B	112.6 (14)
H14A—C14—H14B	112.4 (14)	H40A—C40—H40C	111.2 (15)
H14A—C14—H14C	112.1 (15)	H40B—C40—H40C	107.6 (15)
H14B—C14—H14C	109.7 (15)	O10—C40—H40A	109.3 (10)
O4—C14—H14A	107.6 (11)	O10—C40—H40B	105.3 (11)
O4—C14—H14B	110.9 (10)	O10—C40—H40C	110.7 (11)
O4—C14—H14C	103.8 (12)	O10—C41—C42	112.48 (10)
O4—C15—C16	112.90 (10)	O11—C41—C42	124.42 (11)
O5—C15—C16	124.28 (11)	O11—C41—O10	123.09 (11)
O5—C15—O4	122.82 (11)	C43—C42—C41	120.76 (11)
C17—C16—C15	122.00 (11)	C47—C42—C41	118.26 (11)
C21—C16—C15	117.45 (11)	C47—C42—C43	120.99 (11)
C21—C16—C17	120.54 (11)	C42—C43—H43	117.5 (9)
C16—C17—H17	117.2 (9)	C44—C43—C42	121.41 (11)
C18—C17—C16	121.98 (11)	C44—C43—H43	121.1 (9)
C18—C17—H17	120.8 (9)	C43—C44—C45	118.13 (10)
C17—C18—C19	117.78 (11)	C43—C44—N12	122.95 (10)
C17—C18—N6	122.34 (10)	C45—C44—N12	118.82 (10)
C19—C18—N6	119.88 (10)	C46—C45—C44	119.29 (11)
C20—C19—C18	118.40 (11)	N11—C45—C44	124.85 (10)
N5—C19—C18	126.13 (11)	N11—C45—C46	115.83 (10)
N5—C19—C20	115.46 (10)	C45—C46—S4	109.33 (9)
C19—C20—S2	109.67 (9)	C47—C46—C45	122.60 (11)
C21—C20—C19	123.35 (11)	C47—C46—S4	128.01 (9)
C21—C20—S2	126.97 (9)	C42—C47—H47	121.5 (9)
C16—C21—H21	121.3 (11)	C46—C47—C42	117.57 (11)
C20—C21—C16	117.65 (11)	C46—C47—H47	120.9 (9)
C20—C21—H21	121.0 (11)	N10—C48—S4	119.55 (9)
N4—C22—S2	118.19 (9)	N11—C48—N10	124.09 (11)
N5—C22—N4	125.58 (11)	N11—C48—S4	116.35 (9)
N5—C22—S2	116.10 (9)	H49A—C49—H49B	109.6 (13)
H23A—C23—H23B	108.0 (12)	C50—C49—H49A	109.3 (9)
C24—C23—H23A	109.1 (9)	C50—C49—H49B	110.0 (9)
C24—C23—H23B	109.2 (9)	N12—C49—H49A	110.2 (9)
N6—C23—H23A	110.5 (9)	N12—C49—H49B	108.8 (9)
N6—C23—H23B	109.7 (9)	N12—C49—C50	108.91 (10)
N6—C23—C24	110.32 (10)	C49—C50—H50A	108.3 (10)
C23—C24—H24A	109.2 (8)	C49—C50—H50B	110.5 (9)
C23—C24—H24B	111.7 (9)	H50A—C50—H50B	110.3 (13)
H24A—C24—H24B	110.0 (12)	O12—C50—C49	111.73 (11)
O6—C24—C23	111.30 (9)	O12—C50—H50A	106.5 (10)
O6—C24—H24A	105.7 (8)	O12—C50—H50B	109.5 (9)
O6—C24—H24B	108.7 (9)	H51A—C51—H51B	109.5 (12)
H25A—C25—H25B	109.7 (12)	C52—C51—H51A	110.6 (8)
C26—C25—H25A	110.5 (9)	C52—C51—H51B	111.1 (9)
C26—C25—H25B	110.2 (9)	O12—C51—H51A	108.6 (8)
O6—C25—H25A	109.8 (9)	O12—C51—H51B	106.0 (8)
O6—C25—H25B	105.4 (9)	O12—C51—C52	110.90 (10)
O6—C25—C26	111.10 (10)	C51—C52—H52A	108.9 (9)
C25—C26—H26A	108.7 (9)	C51—C52—H52B	110.0 (9)
C25—C26—H26B	108.6 (9)	H52A—C52—H52B	109.3 (13)
H26A—C26—H26B	107.7 (13)	N12—C52—C51	109.03 (9)
N6—C26—C25	110.90 (10)	N12—C52—H52A	110.0 (9)
N6—C26—H26A	108.1 (9)	N12—C52—H52B	109.5 (9)
N6—C26—H26B	112.8 (9)	C48—N10—H10C	118.6 (11)
C22—N4—H4A	120.7 (12)	C48—N10—H10D	118.3 (12)
C22—N4—H4B	115.9 (12)	H10C—N10—H10D	120.8 (16)
H4A—N4—H4B	119.6 (16)	C48—N11—C45	110.03 (10)
C22—N5—C19	110.04 (10)	C44—N12—C49	114.59 (9)
C18—N6—C23	113.44 (9)	C44—N12—C52	115.29 (9)
C18—N6—C26	114.89 (9)	C52—N12—C49	109.40 (9)
C26—N6—C23	109.89 (9)	C41—O10—C40	115.89 (10)
C15—O4—C14	115.72 (10)	C50—O12—C51	109.81 (9)
C24—O6—C25	109.36 (9)	C46—S4—C48	88.47 (6)
C20—S2—C22	88.63 (6)	H1WA—O1W—H1WB	106.1 (18)
H27A—C27—H27B	109.0 (13)		
			
C2—C3—C4—C5	179.69 (11)	C28—C29—C30—C31	−178.77 (11)
C2—C3—C8—C7	−178.65 (10)	C28—C29—C34—C33	178.59 (10)
C3—C2—O1—C1	−179.88 (11)	C29—C28—O7—C27	175.96 (10)
C3—C4—C5—C6	−2.03 (17)	C29—C30—C31—C32	0.99 (17)
C3—C4—C5—N3	179.16 (10)	C29—C30—C31—N9	−175.16 (11)
C4—C3—C8—C7	2.59 (17)	C30—C29—C34—C33	−1.23 (17)
C4—C5—C6—C7	4.50 (16)	C30—C31—C32—C33	−2.75 (17)
C4—C5—C6—N2	−173.70 (11)	C30—C31—C32—N8	−179.89 (11)
C4—C5—N3—C10	−121.63 (12)	C30—C31—N9—C36	−117.24 (13)
C4—C5—N3—C13	5.38 (15)	C30—C31—N9—C39	14.01 (17)
C5—C6—C7—C8	−3.65 (17)	C31—C32—C33—C34	2.66 (18)
C5—C6—C7—S1	179.19 (8)	C31—C32—C33—S3	−176.51 (9)
C5—C6—N2—C9	179.27 (11)	C31—C32—N8—C35	176.70 (11)
C6—C5—N3—C10	59.59 (14)	C32—C31—N9—C36	66.67 (14)
C6—C5—N3—C13	−173.40 (10)	C32—C31—N9—C39	−162.08 (11)
C6—C7—C8—C3	0.03 (18)	C32—C33—C34—C29	−0.62 (18)
C6—C7—S1—C9	2.31 (9)	C32—C33—S3—C35	−0.71 (9)
C7—C6—N2—C9	1.01 (14)	C33—C32—N8—C35	−0.53 (15)
C8—C3—C4—C5	−1.60 (18)	C34—C29—C30—C31	1.04 (18)
C8—C7—S1—C9	−174.71 (11)	C34—C33—S3—C35	−179.82 (12)
C10—C11—O3—C12	60.51 (12)	C36—C37—O9—C38	59.29 (14)
C11—C10—N3—C5	−177.19 (9)	C37—C36—N9—C31	−171.23 (10)
C11—C10—N3—C13	53.45 (12)	C37—C36—N9—C39	54.89 (13)
C12—C13—N3—C5	176.77 (10)	C38—C39—N9—C31	169.60 (10)
C12—C13—N3—C10	−54.68 (12)	C38—C39—N9—C36	−56.83 (14)
C13—C12—O3—C11	−61.98 (12)	C39—C38—O9—C37	−61.82 (13)
N1—C9—N2—C6	−178.42 (11)	N7—C35—N8—C32	179.54 (11)
N1—C9—S1—C7	177.42 (10)	N7—C35—S3—C33	−179.15 (10)
N2—C6—C7—C8	174.74 (11)	N8—C32—C33—C34	−179.96 (11)
N2—C6—C7—S1	−2.42 (13)	N8—C32—C33—S3	0.87 (13)
N2—C9—S1—C7	−1.94 (9)	N8—C35—S3—C33	0.47 (10)
N3—C5—C6—C7	−176.66 (10)	N9—C31—C32—C33	173.56 (10)
N3—C5—C6—N2	5.14 (17)	N9—C31—C32—N8	−3.58 (17)
N3—C10—C11—O3	−56.98 (12)	N9—C36—C37—O9	−56.01 (14)
O1—C2—C3—C4	−8.17 (16)	O7—C28—C29—C30	−10.70 (16)
O1—C2—C3—C8	173.08 (10)	O7—C28—C29—C34	169.49 (10)
O2—C2—C3—C4	172.21 (12)	O8—C28—C29—C30	168.63 (12)
O2—C2—C3—C8	−6.54 (18)	O8—C28—C29—C34	−11.18 (19)
O2—C2—O1—C1	−0.25 (17)	O8—C28—O7—C27	−3.40 (17)
O3—C12—C13—N3	60.09 (13)	O9—C38—C39—N9	60.91 (14)
S1—C7—C8—C3	176.68 (9)	S3—C33—C34—C29	178.38 (9)
S1—C9—N2—C6	0.90 (13)	S3—C35—N8—C32	−0.06 (13)
C15—C16—C17—C18	−178.95 (11)	C41—C42—C43—C44	−179.96 (10)
C15—C16—C21—C20	179.19 (11)	C41—C42—C47—C46	178.85 (10)
C16—C15—O4—C14	−179.39 (10)	C42—C41—O10—C40	−179.97 (10)
C16—C17—C18—C19	1.38 (17)	C42—C43—C44—C45	0.88 (17)
C16—C17—C18—N6	−178.92 (11)	C42—C43—C44—N12	−175.40 (10)
C17—C16—C21—C20	−2.33 (17)	C43—C42—C47—C46	−1.07 (17)
C17—C18—C19—C20	−5.46 (16)	C43—C44—C45—C46	−0.57 (16)
C17—C18—C19—N5	176.13 (11)	C43—C44—C45—N11	−178.47 (10)
C17—C18—N6—C23	122.69 (12)	C43—C44—N12—C49	−116.36 (12)
C17—C18—N6—C26	−4.90 (16)	C43—C44—N12—C52	11.97 (16)
C18—C19—C20—C21	6.00 (17)	C44—C45—C46—C47	−0.58 (17)
C18—C19—C20—S2	−175.03 (9)	C44—C45—C46—S4	−177.96 (9)
C18—C19—N5—C22	175.08 (11)	C44—C45—N11—C48	177.41 (11)
C19—C18—N6—C23	−57.61 (14)	C45—C44—N12—C49	67.40 (13)
C19—C18—N6—C26	174.79 (10)	C45—C44—N12—C52	−164.28 (10)
C19—C20—C21—C16	−2.02 (18)	C45—C46—C47—C42	1.39 (17)
C19—C20—S2—C22	−2.05 (9)	C45—C46—S4—C48	0.23 (9)
C20—C19—N5—C22	−3.37 (14)	C46—C45—N11—C48	−0.56 (14)
C21—C16—C17—C18	2.65 (18)	C47—C42—C43—C44	−0.05 (18)
C21—C20—S2—C22	176.87 (11)	C47—C46—S4—C48	−176.96 (12)
C23—C24—O6—C25	−59.78 (12)	C49—C50—O12—C51	58.78 (14)
C24—C23—N6—C18	175.00 (10)	C50—C49—N12—C44	−171.17 (10)
C24—C23—N6—C26	−54.85 (12)	C50—C49—N12—C52	57.59 (13)
C25—C26—N6—C18	−175.84 (10)	C51—C52—N12—C44	170.33 (10)
C25—C26—N6—C23	54.79 (12)	C51—C52—N12—C49	−58.80 (12)
C26—C25—O6—C24	59.25 (12)	C52—C51—O12—C50	−59.47 (13)
N4—C22—N5—C19	177.51 (11)	N10—C48—N11—C45	179.56 (11)
N4—C22—S2—C20	−175.92 (10)	N10—C48—S4—C46	−179.45 (10)
N5—C19—C20—C21	−175.42 (11)	N11—C45—C46—C47	177.50 (11)
N5—C19—C20—S2	3.54 (13)	N11—C45—C46—S4	0.13 (13)
N5—C22—S2—C20	0.25 (9)	N11—C48—S4—C46	−0.59 (10)
N6—C18—C19—C20	174.83 (10)	N12—C44—C45—C46	175.87 (10)
N6—C18—C19—N5	−3.57 (18)	N12—C44—C45—N11	−2.04 (17)
N6—C23—C24—O6	58.12 (13)	N12—C49—C50—O12	−57.99 (14)
O4—C15—C16—C17	−1.46 (16)	O10—C41—C42—C43	6.08 (15)
O4—C15—C16—C21	177.00 (10)	O10—C41—C42—C47	−173.84 (10)
O5—C15—C16—C17	178.62 (12)	O11—C41—C42—C43	−174.56 (11)
O5—C15—C16—C21	−2.92 (18)	O11—C41—C42—C47	5.52 (18)
O5—C15—O4—C14	0.53 (17)	O11—C41—O10—C40	0.67 (17)
O6—C25—C26—N6	−57.66 (13)	O12—C51—C52—N12	60.01 (13)
S2—C20—C21—C16	179.19 (9)	S4—C46—C47—C42	178.24 (9)
S2—C22—N5—C19	1.66 (12)	S4—C48—N11—C45	0.75 (13)

### Methyl 2-amino-4-(morpholin-4-yl)benzo[d]thiazole-6-carboxylate tetartohydrate Hydrogen-bond geometry (Å, º)

**Table:**

*D*—H···*A*	*D*—H	H···*A*	*D*···*A*	*D*—H···*A*
C11—H11*B*···O6^i^	0.994 (15)	2.497 (15)	3.4101 (15)	152.5 (12)
N1—H1*D*···N5	0.844 (18)	2.324 (18)	3.1642 (15)	174.3 (16)
N1—H1*E*···O11^ii^	0.876 (18)	2.137 (18)	2.9475 (14)	153.6 (15)
C24—H24*A*···O3^iii^	1.010 (15)	2.638 (15)	3.5311 (15)	147.4 (11)
C24—H24*B*···O11^ii^	1.009 (16)	2.591 (16)	3.4912 (15)	148.4 (12)
C25—H25*A*···N8^iv^	0.972 (15)	2.655 (15)	3.4047 (16)	134.2 (11)
C25—H25*B*···N8^iii^	0.964 (15)	2.603 (16)	3.4115 (16)	141.6 (12)
N4—H4*A*···N2	0.90 (2)	2.13 (2)	2.9881 (15)	159.0 (16)
N4—H4*B*···O1*W*^v^	0.858 (19)	1.957 (19)	2.8038 (14)	168.8 (17)
C34—H34···O1*W*^i^	0.950 (16)	2.580 (16)	3.4294 (15)	149.0 (12)
N7—H7*A*···O2^vi^	0.840 (19)	2.024 (19)	2.8556 (14)	170.8 (17)
N7—H7*B*···O6^iv^	0.857 (19)	2.120 (19)	2.9625 (14)	167.5 (17)
C40—H40*A*···N7^iii^	0.981 (18)	2.691 (17)	3.5283 (18)	143.5 (13)
N10—H10*C*···O5^iii^	0.870 (18)	2.088 (18)	2.9101 (14)	157.3 (15)
N10—H10*D*···O3^v^	0.839 (19)	2.11 (2)	2.9479 (14)	172.6 (17)
O1*W*—H1*WA*···O8^iii^	0.93 (2)	1.84 (2)	2.7451 (13)	162.6 (19)
O1*W*—H1*WB*···N12	0.97 (2)	1.91 (2)	2.8669 (13)	169 (2)

Symmetry codes: (i) *x*+1, *y*, *z*; (ii) −*x*, −*y*+1, −*z*+1; (iii) *x*−1, *y*, *z*; (iv) −*x*+1, −*y*+1, −*z*+1; (v) −*x*+1, −*y*, −*z*+1; (vi) *x*+1, *y*, *z*−1.
